# Searching for New Biomarkers of Neuroendocrine Tumors: A Comparative Analysis of Chromogranin A and Inflammatory Cytokines in Patients with Neuroendocrine Tumors

**DOI:** 10.3390/curroncol31100456

**Published:** 2024-10-12

**Authors:** Marlena Budek, Jarosław Nuszkiewicz, Jolanta Czuczejko, Marta Maruszak-Parda, Joanna Wróblewska, Jakub Wojtasik, Iga Hołyńska-Iwan, Marta Pawłowska, Alina Woźniak, Karolina Szewczyk-Golec

**Affiliations:** 1 Department of Medical Biology and Biochemistry, Faculty of Medicine, Ludwik Rydygier Collegium Medicum in Bydgoszcz, Nicolaus Copernicus University in Toruń, 87-100 Toruń, Poland; marlenamarkiewicz@o2.pl (M.B.); jnuszkiewicz@cm.umk.pl (J.N.); joanna.wroblewska@cm.umk.pl (J.W.); marta.pawlowska@cm.umk.pl (M.P.); al1103@cm.umk.pl (A.W.); 2 Department of Psychiatry, Faculty of Medicine, Ludwik Rydygier Collegium Medicum in Bydgoszcz, Nicolaus Copernicus University in Toruń, 87-100 Toruń, Poland; joczu@cm.umk.pl; 3 Department of Nuclear Medicine, Oncology Centre Prof. Franciszek Łukaszczyk Memorial Hospital, 85-796 Bydgoszcz, Poland; maruszakm@co.bydgoszcz.pl; 4 Centre for Statistical Analysis, Nicolaus Copernicus University in Toruń, 87-100 Toruń, Poland; jwojtasik@umk.pl; 5 Department of Pathobiochemistry and Clinical Chemistry, Faculty of Pharmacy, Ludwik Rydygier Collegium Medicum in Bydgoszcz, Nicolaus Copernicus University in Torun, 87-100 Toruń, Poland; igaholynska@cm.umk.pl

**Keywords:** cancer, chromogranin A, cytokines, diagnostic markers, neuroendocrine tumors

## Abstract

Neuroendocrine neoplasms (NENs) present a diagnostic challenge due to their heterogeneous nature and non-specific clinical manifestations. This study aimed to explore novel biomarkers for NENs. Serum chromogranin A (CgA) levels and a panel of 48 inflammatory cytokines were analyzed in a cohort of 84 NEN patients and 40 healthy controls using enzyme-linked immunosorbent assay (ELISA) and multiplex ELISA. Significant alterations in cytokine levels were observed in the NEN patients compared to the controls, including elevated levels of pro-inflammatory cytokines, such as interleukin (IL)-6, IL-8, and tumor necrosis factor alpha (TNF-α), and reduced levels of angiogenic factors like platelet-derived growth factor-BB (PDGF-BB) and tumor necrosis factor beta (TNF-β). Notably, cytokines such as growth-regulated alpha protein (GRO-α) and TNF-β demonstrated strong potential as diagnostic markers, with receiver operating characteristic (ROC) curve analyses showing high sensitivity and specificity. Additionally, a positive correlation was found between CgA levels and several inflammatory cytokines, suggesting their synergistic role in tumor progression. These findings highlight the limited reliability of CgA alone as a diagnostic marker and underscore the importance of a multi-marker approach in diagnosing and monitoring NENs. Further research on a larger cohort is necessary to validate these biomarkers and their potential clinical applications.

## 1. Introduction

Neuroendocrine neoplasms (NENs) constitute a heterogeneous group of tumors [1]. They are characterized by the ability to produce, store, and secrete bioactive peptides and biogenic amines in response to neural, chemical, and other stimuli [2]. Epidemiological data show that primary tumors are most often located in the digestive system and pancreas (62–67%) and in the bronchopulmonary system (22–27%); NENs with metastatic disease constitute 12–22% [3].

The diagnosis of NENs is complex and often ambiguous due to their heterogeneous clinical manifestations, suggesting the presence of other disease entities, including non-cancerous ones [4]. However, significant progress has been made in the field of imaging diagnostics of NENs, also by combining anatomical and functional methods, which significantly increased the sensitivity of their use [4]. In addition to improving imaging methods, which are the most useful indicator of the disease progression, attempts are still being made to identify markers determined in blood serum that could be helpful in the diagnosis of NENs, assessment of treatment effectiveness, and the determination of the stage of tumor advancement [5]. A particular diagnostic problem includes hereditary multiple endocrine neoplasia (MEN), in which case the diagnosis must be based on a multidisciplinary approach to the patient [6]. Early diagnosis of MEN should focus mainly on genetic tests and rapid screening tests, which could be based on biochemical methods, because radiological tests are not always effective, e.g., in the case of pancreatic neuroendocrine tumors (even about 50% of undetected cases) [7]. NETest (the neuroendocrine neoplasm test), which is a multi-analyte liquid biopsy measuring the expression of NEN genes in the blood, is gaining great popularity and is already used in clinical practice [8]. The assessment of circulating microRNAs specific to a given tumor precisely determines the metabolic activity of a single tumor in real time and has clinical application in three levels, namely in assessing the effectiveness of surgical treatment, in assessing the response to the treatment with somatostatin analogs and peptide receptor radionuclide therapy, as well as in predicting the disease recurrence and progression [9]. The parameters most frequently used in the diagnosis of NENs include chromogranin A (CgA), neurospecific enolase (NSE), pancreatic polypeptide (PP), and hormones released by tumors, such as insulin, gastrin, glucagon, or somatostatin, at the same time confirming their clinical symptoms [10]. Due to the heterogeneity of the studied group of cancers, finding a reliable, universal marker for all studied locations is particularly difficult, and those known and used so far have limited predictive and prognostic value [8]. There is a need to identify a parameter or a panel of parameters that would identify NENs of various locations, due to, for example, the asymptomatic occurrence of tumors or co-occurrence of tumors in many organs simultaneously, e.g., in MEN. CgA is one of the substances that is co-stored and secreted by exocytosis together with resident catecholamines, peptide hormones, or neurotransmitters specific to given neuroendocrine cells, e.g., CgA is released from enterochromaffin cells together with serotonin [11]. Data in the literature show that the simultaneous measurement of CgA and NSE shows higher sensitivity and greater accuracy in predicting the prognosis and progression of the disease than the measurement of each of these parameters separately [12,13].

Undoubtedly, in the search for easily accessible diagnostic biomarkers of NENs, the tumor microenvironment (TME) deserves attention as it contains many types of cells and factors, including growth factors, cytokines, and chemokines, which regulate the immune response via numerous signaling pathways [14]. Systemic inflammation may cause excessive stimulation of neuroendocrine cells, leading to hyperplasia and neoplastic transformation; however, the appropriate modulation of the immune system may constitute a therapeutic target. Moreover, inflammatory markers may be a promising tool for use in diagnosis, prediction of response to treatment, and as prognostic biomarkers in NENs [15,16]. According to the research by Papalou et al. [17], there is a positive correlation of CgA with C-reactive protein (CRP) in patients with NENs. Interestingly, as the CRP concentration increases, there is a greater likelihood of NENs with metastases than without metastases [17]. The process of carcinogenesis at each stage is also associated with chronic oxidative stress, which may induce chronic inflammation. As a result, mediators can change cell signaling, inducing changes in the level of transcription factors, e.g., nuclear factor kappa B (NF-кB), signal transducer and activator of transcription 3 (STAT3), hypoxia-inducible factor-1 (HIF1-α), and the abnormal expression of cytokines and inflammatory chemokines, e.g., tumor necrosis factor (TNF), interleukins (IL): IL-1, IL-6, IL-8, or chemokine CXC motif receptor 4 (CXCR4) [18].

The diagnostic process of NENs is not obvious, and the challenge for clinicians is particularly posed by hormonally inactive NENs, i.e., neuroendocrine tumors that do not manifest clinical symptoms. It should be noted that NENs have not been the subject of many studies in the context of inflammation. Therefore, the aim of this study was to search for links between inflammation and the development of NENs and to identify biomarkers circulating in the blood in patients with newly diagnosed NENs. In addition to determining the parameters with the highest sensitivity useful in the diagnosis of NENs, the aim of the study was also to determine the correlation between these parameters and CgA, and to determine the probability of disease occurrence based on the tested analytes.

## 2. Materials and Methods

### 2.1. Study Subjects

The study was conducted on a group of 84 patients with NENs and 40 healthy people constituting the control group. Patients of the Oncology Center of the Prof. Franciszek Łukaszczyk Memorial Hospital located in Bydgoszcz, Poland, due to the suspicion of neuroendocrine tumors, were subjected to radioisotope diagnostics in order to confirm or exclude the diagnosis, locate the primary lesion, and determine the type of NEN or locate metastases. The condition for including the patient in the study was the diagnosis of neuroendocrine cancer. The patient exclusion criteria included the presence of other acute and chronic diseases. The study group consisted of the patients with tumors located in the pancreas—pancreatic neuroendocrine neoplasms (pNENs, *n* = 22); gastrointestinal tract—gastrointestinal neuroendocrine neoplasms (GI-NENs, *n* = 32); lung—lung neuroendocrine neoplasms (L-NENs, *n* = 12); and other previously unclassified locations—other neuroendocrine neoplasms (o-NENs, *n* = 18). The analyzed cases were primary tumors without evident metastatic processes. Based on the patient history, carcinoid syndrome was documented in only one case of GI-NENs. According to the 2019 classification by the International Agency for Research on Cancer (IARC) under the World Health Organization (WHO), the analyzed cases were classified as well-differentiated tumors with low-grade malignancy (NET G1) and intermediate-grade malignancy (NET G2). The control group consisted of volunteers. The exclusion criteria for the control group included chronic and/or acute diseases such as cancer, diabetes, obesity, autoimmune diseases, and cardiometabolic disorders. The consent to perform the study was obtained from the Bioethics Committee of the Nicolaus Copernicus University in Toruń, functioning at the Collegium Medicum in Bydgoszcz, Poland (consent No. KB 423/2020 approved on 29 September 2020). The project participants were informed about the purpose of the research and provided written consent to participate in the experiment. The results of anthropometric analyzes are presented in Table 1.

### 2.2. Study Design

Blood samples were collected in the morning, after an overnight fast, between 7:00 a.m. and 9:00 a.m., from the median cubital vein. This procedure was conducted by qualified medical personnel at the Department of Nuclear Medicine, Oncology Center, Hospital Prof. Franciszek Łukaszczyk in Bydgoszcz, Poland. The blood was drawn into 9 mL polypropylene tubes containing a clotting activator and a gel separator.

Immediately after collection, the samples were transported under reduced temperature conditions to the laboratory of the Department of Medical Biology and Biochemistry, Faculty of Medicine, Ludwik Rydygier Collegium Medicum in Bydgoszcz, Nicolaus Copernicus University in Toruń, Poland. The blood samples were centrifuged at 6000× *g* for 10 min at 4 °C to separate the serum from the blood clot. Following centrifugation, the serum was divided into aliquots and stored at −80 °C until further biochemical analysis.

### 2.3. Biochemical Analysis

The concentration of CgA in the blood serum was measured using an enzyme-linked immunosorbent assay (ELISA) kit from Cloud-Clone Corp. (Katy, TX, USA). The readings were obtained using a SPECTROstar Nano plate spectrophotometer (BMG LABTECH, Ortenberg, Germany).

The concentrations of 48 inflammatory parameters were determined using the Bio-Plex Pro Human Cytokine Screening Panel, 48-plex (Bio-Rad Laboratories Inc., Hercules, CA, USA). This multiplex ELISA test was read using the Bio-Plex System 200 analyzer (Bio-Rad Laboratories Inc., Hercules, CA, USA). The Bio-Plex Pro Human Cytokine Screening Panel measures a comprehensive array of biomarkers, including cutaneous T-cell-attracting chemokine (CTACK), eosinophil chemotactic protein (Eotaxin), basic fibroblast growth factor (bFGF), granulocyte colony-stimulating factor (G-CSF), granulocyte-macrophage colony-stimulating factor (GM-CSF), growth-regulated alpha protein (GRO-α), hepatocyte growth factor (HGF), interferon alpha-2 (IFN-α2), interferon gamma (IFN-γ), interleukin 1 alpha (IL-1α), interleukin 1 beta (IL-1β), interleukin 1 receptor antagonist (IL-1ra), interleukin 2 (IL-2), interleukin 2 receptor alpha (IL-2Rα), interleukin 3 (IL-3), interleukin 4 (IL-4), interleukin 5 (IL-5), interleukin 6 (IL-6), interleukin 7 (IL-7), interleukin 8 (IL-8), interleukin 9 (IL-9), interleukin 10 (IL-10), interleukin 12 (p70) (IL-12 (p70)), interleukin 12 (p40) (IL-12 (p40)), interleukin 13 (IL-13), interleukin 15 (IL-15), interleukin 16 (IL-16), interleukin 17A (IL-17A), interleukin 18 (IL-18), interferon gamma-induced protein 10 (IP-10), leukemia inhibitory factor (LIF), monocyte chemoattractant protein-1 (MCP-1), monocyte chemoattractant protein-3 (MCP-3), macrophage colony-stimulating factor (M-CSF), macrophage migration inhibitory factor (MIF), monokine induced by gamma interferon (MIG), macrophage inflammatory protein-1 alpha (MIP-1α), macrophage inflammatory protein-1 beta (MIP-1β), beta-nerve growth factor (β-NGF), platelet-derived growth factor-BB (PDGF-BB), regulated on activation, normal T cell expressed and secreted (RANTES), stem cell factor (SCF), stem cell growth factor beta (SCGF-β), stromal cell-derived factor 1 alpha and beta (SDF-1α+β), tumor necrosis factor alpha (TNF-α), tumor necrosis factor beta (TNF-β), TNF-related apoptosis-inducing ligand (TRAIL), and vascular endothelial growth factor (VEGF). These analytes were determined using a commercially available research kit, following the manufacturer’s instructions for all analyses. The enzyme immune assay kit included standard concentration analytes, blank and control samples, and other necessary reagents. Bio-Plex Multiplex immunoassay is a technique used for the detection and quantification of multiple protein biomarkers simultaneously in a single sample. It involves the use of magnetic beads coated with specific antibodies to capture and detect different proteins in a complex mixture. For the analysis, a serum volume of 15 µL was used, requiring at least 50 magnetic beads in each region to complete the sample analysis. The proteins captured by the beads bind to detection antibodies, forming a “sandwich” complex, which is then targeted by a streptavidin–phycoerythrin (SA-PE) conjugate, introducing fluorescence through phycoerythrin as a fluorescent indicator. The next step involves adding a reporter dye to the reaction mixture, followed by reading using a laser-based system that detects the emitted fluorescence. Fluorescence was measured using the Bio-Plex^®^ 200 system, which employs dual-laser technology for precise measurement. A red laser (635 nm) identifies each bead, determining the analyte based on the bead’s fluorescence, while a green laser (532 nm) quantifies the reporter signal from the phycoerythrin, correlating directly with the analyte’s concentration. This process is managed by a high-speed digital processor, and results are presented using Bio-Plex Manager™ 6.2 Software as median fluorescence intensity (MFI), allowing for the accurate determination of concentrations based on the MFI. To ensure accuracy and reliability, each sample concentration underwent duplicate measurements, and the average of the two measurements was included in the analysis. The results were reported in picograms per milliliter (pg/mL) or nanograms per milliliter (ng/mL).

### 2.4. Statistical Analysis

The statistical analysis was performed using the R Statistical Software v4.3.1 by R Foundation for Statistical Computing—R Core Team 2023 (Vienna, Austria) and Python 3.8.10 from the Python Software Foundation (Wilmington, DE, USA), incorporating libraries such as statsmodels (0.13.5), matplotlib (3.6.3), pandas (1.4.3), and scikit learn (1.1.3). The data were presented as mean values, standard error of the mean (SEM), medians, and the lower (Q_1_) and upper quartiles (Q_3_). The normality of the data distributions was verified for each variable using the Shapiro–Wilk test. If the assumption of normality was rejected, the Mann–Whitney U test was performed, and conclusions were based on the medians. The significance level was set at *p* < 0.05. Pearson’s correlation coefficients were calculated to determine the relationships between CgA values and other examined parameters. Additionally, receiver operating characteristic (ROC) curve analysis was performed for selected parameters to describe the relationship between detected neuroendocrine neoplasms (NENs) and the values of the tested parameters. This included a multivariable logistic regression model to determine the probability of disease occurrence in a patient based on the values of GRO-α and TNF-β, assessing their usefulness as diagnostic parameters for NENs. Cut-off points for variables were determined using the Youden index to optimize the sensitivity and specificity of the parameters.

## 3. Results

### 3.1. Serum Levels of Cytokines and CgA Are Altered in Patients with Neuroendocrine Tumors

Among the inflammatory parameters, a statistically significant increase in concentrations was observed in the study group compared to the control group for CTACK, Eotaxin, bFGF, G-CSF, GM-CSF, GRO-α, HGF, IFN-α2, IFN-γ, IL-1α, IL-1β, IL-1ra, IL-2, IL-2Rα, IL-4, IL-5, IL-6, IL-7, IL-8, IL-10, IL-15, IL-16, IL-17A, IL-18, LIF, MCP-1, MCP-3, M-CSF, MIF, MIG, MIP-1α, β-NGF, RANTES, SCF, SCGF-β, TNF-α, TRAIL, and VEGF. A statistically significant decrease in concentrations was observed for PDGF-BB, MIP-1β, SDF-1α+β, and TNF-β. There were no statistically significant changes in IL-9, IL-13, and IP-10 in the study group. Statistically significant changes were also noted for IL-3, IL-12 (p40), and IL-12 (p70). A summary of the presented results is provided in Table 2.

### 3.2. Correlation between CgA and Selected Cytokines

**Figure 1:**
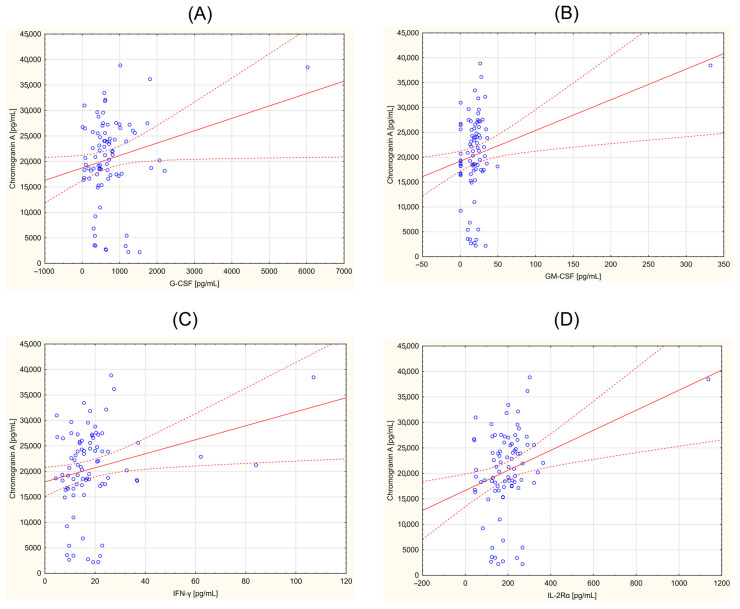
Correlation between chromogranin A (CgA) and selected cytokines in a group of the patients with neuroendocrine tumors. (**A**)—granulocyte colony-stimulating factor (G-CSF) vs. CgA (r = 0.219; *p* = 0.045); (**B**)—granulocyte-macrofage colony-stimulating factor (GM-CSF) vs. CgA (r = 0.267; *p* = 0.014); (**C**)—interferone gamma (IFN-γ) vs. CgA (r = 0.248; *p* = 0.023); (**D**)—interleukin 2 receptor subunit alpha (IL-2Rα) vs. CgA (r = 0.301; *p* = 0.005).

**Figure 2:**
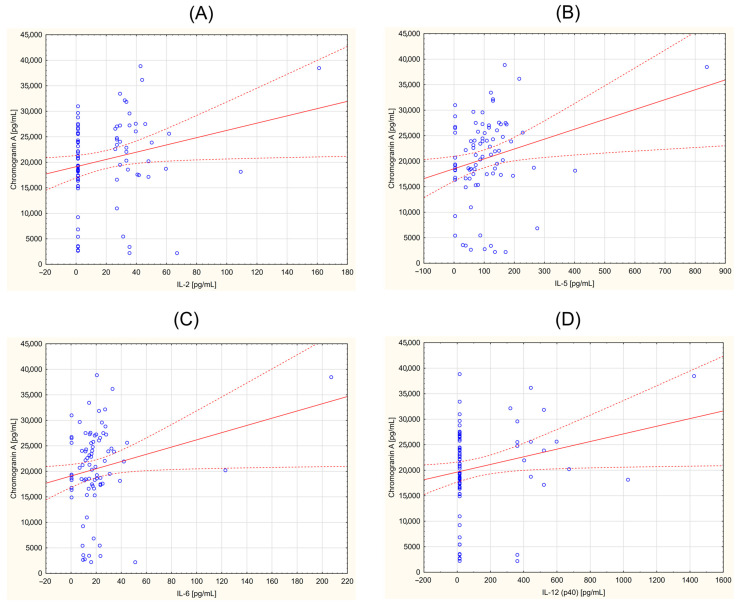
Correlation between chromogranin A (CgA) and selected cytokines in a group of the patients with neuroendocrine tumors. (**A**)—interleukin 2 (IL2) vs. CgA (r = 0.228; *p* = 0.037); (**B**)—interleukin 5 (IL-5) vs. CgA (r = 0.254; *p* = 0.020); (**C**)—Interleukin 6 (IL-6) vs. CgA (r = 0.221; *p* = 0.043), (**D**)—interleukin IL-12 (p40) vs. CgA (r = 0.222; *p* = 0.042).

**Figure 3:**
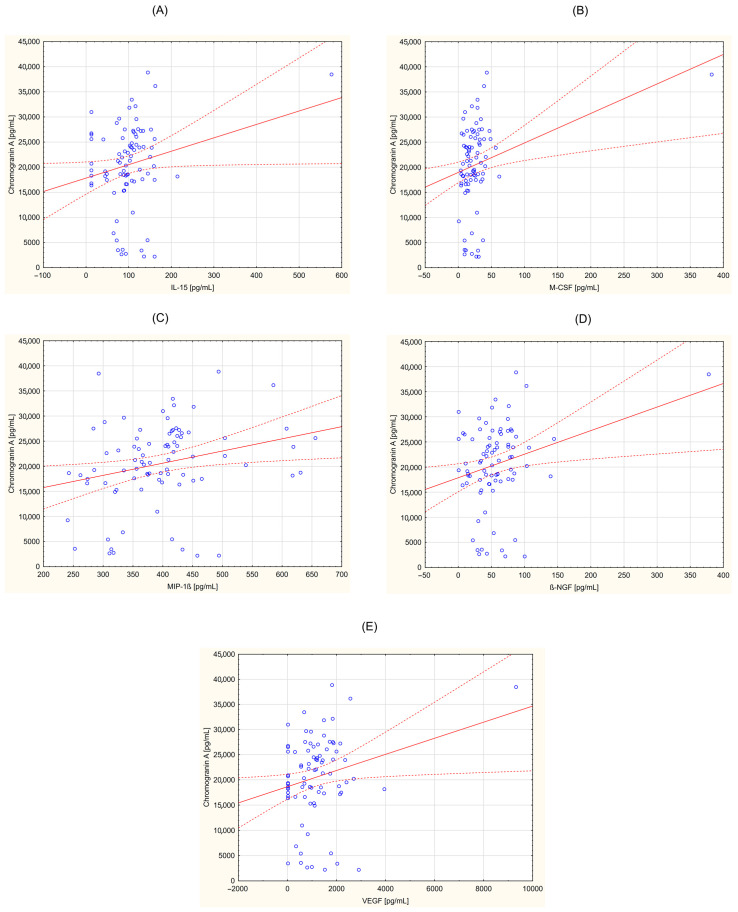
Correlation between chromogranin A (CgA) and selected cytokines in a group of patients with neuroendocrine tumors. (**A**)—interleukin 15 (IL-15) vs. CgA (r = 0.217; *p* = 0.047); (**B**)—macrophage colony stimulating factor (M-CSF) vs. CgA (r = 0.293; *p* = 0.007); (**C**)—macrophage inflammatory protein 1 beta (MIP-1β) vs. CgA (r = 0.259; *p* = 0.017); (**D**)—nerve growth factor β (β-NGF vs. CgA (r = 0.261; *p* = 0.016); (**E**)—vascular endothelial growth factor (VEGF) vs. CgA (r = 0.234, *p* = 0.032).

The obtained data were also analyzed for correlations between the concentration of inflammatory parameters and CgA in individuals in the study group. The correlation coefficient (r) and statistical significance (*p* < 0.05) were determined. A positive correlation was demonstrated for the following parameters: G-CSF (r = 0.219; *p* = 0.045), GM-CSF (r = 0.267; *p* = 0.014), IFN-γ (r = 0.248; *p* = 0.023), IL-2 (r = 0.228; *p* = 0.037), IL-2Ra (r = 0.301; *p* = 0.005), IL-5 (r = 0.254; *p* = 0.020), IL-6 (r = 0.221; *p* = 0.043), IL-12 (p40) (r = 0.222; *p* = 0.042), IL-15 (r = 0.217; *p* = 0.047), M-CSF (r = 0.293; *p* = 0.007), MIP-1β (r = 0.259; *p* = 0.017), β-NGF (r = 0.261; *p* = 0.016), VEGF (r = 0.234, *p* = 0.032). Figure 1, Figure 2 and Figure 3 provide the Pearson’s correlation diagrams for these parameters within the study group, offering a visual representation of the observed statistical relationships.

### 3.3. Analysis of Serum Cytokines in Healthy Control and Patients with NETs. Receiver Operating Characteristic (ROC)/Area under the Curve (AUC) Was Used to Indicate a Diagnostic Marker with High Sensitivity and Specificity

**Figure 4:**
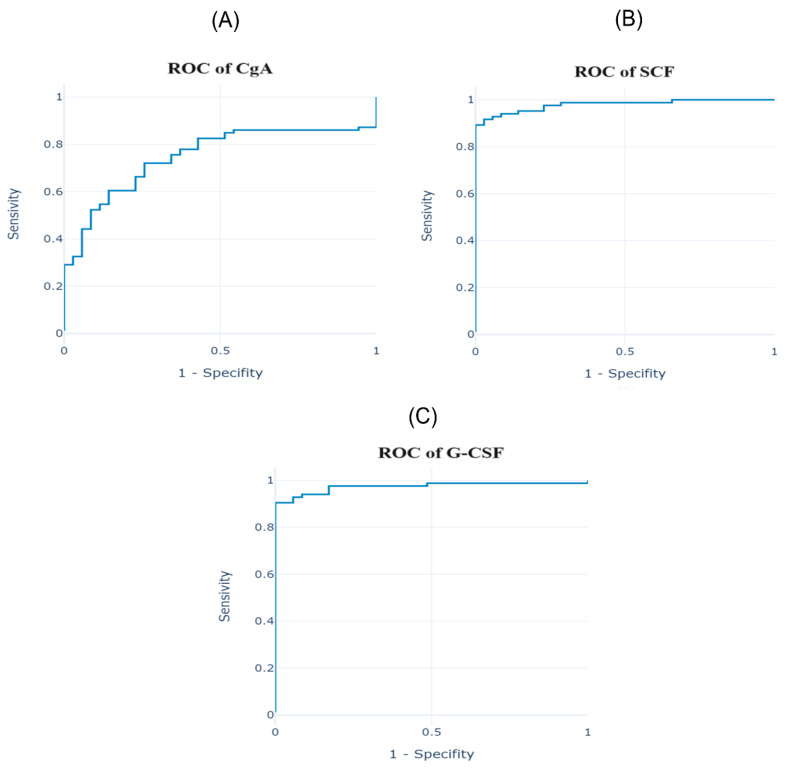

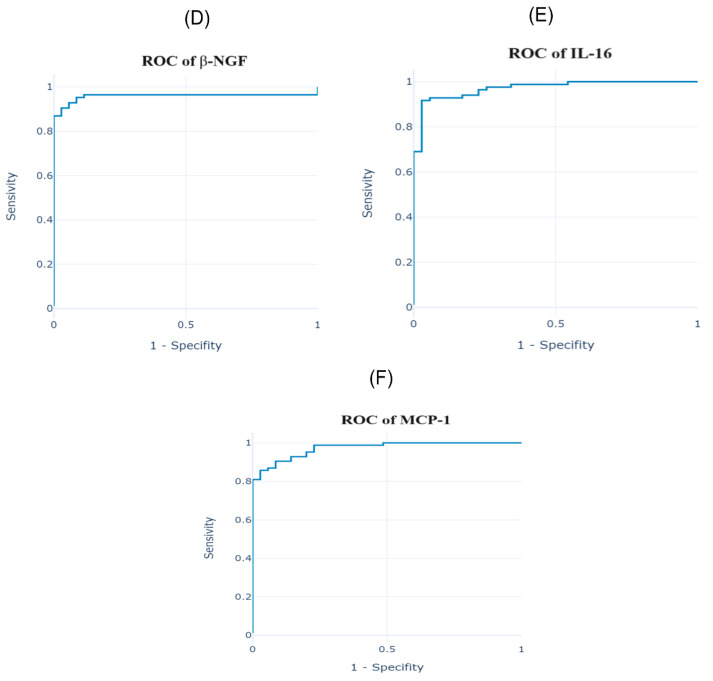
Receiver operating characteristic (ROC) of serum cytokines of the patients diagnosed with neuroendocrine neoplasms (NENs). (**A**)—ROC of chromogranin A (CgA); (**B**)—ROC of stem cell factor (SCF); (**C**)—ROC of granulocyte colony-stimulating factor (G-CSF); (**D**)—ROC of nerve growth factor β (β-NGF); (**E**)—ROC of interleukin 16 (IL-16); (**F**)—ROC of monocyte chemotactic protein 1 (MCP-1).

For parameters showing statistically significant differences, ROC curves were plotted (Figure 4) to identify the potentially most sensitive and specific NENs markers. These curves are presented for parameters with an area under the curve (AUC) greater than 0.9 and *p* < 0.05. These parameters include SCF, G-CSF, β-NGF, IL-16, MCP-1, IL-2Rα, TNF-α, MIF, IL-1α, IL-4, LIF, TNF-β, IL-1ra, IL-1β, IL-15, IL-7, IL-8, IL-6, GRO-α, M-CSF, CTACK, SCGF-β, and IFN-α2. Figure 4 highlights the parameters with the highest AUC values (>0.95). For these variables, Table 3 presents the AUC values, confidence intervals, *p*-values, and designated cut-off points (pg/mL).

The analysis showed that TNF-β concentration is a negative marker, meaning lower values allow for the classification of a patient as having the disease. For the remaining parameters described, higher values indicate a predisposition to the detection of the disease. Additionally, the ROC curve for CgA, the most frequently used parameter in NENs, is also graphically presented below.

The remaining parameters showing statistically significant changes in the ROC curve analysis, with AUC less than 0.9, include HGF, IFN-γ, GM-CSF, VEGF, MCP-3, IL-5, IL-10, IL-17A, RANTES, MIG, PDGF-BB, Eotaxin, CgA, IL-2, MIP-1α, TRAIL, bFGF, IL-12 (p70), IL-18, IL-3, and IL-12 (p40). There were no statistically significant changes in the following variables: MIP-1β, IL-9, IP-10, SDF-1α+β, and IL-13.

### 3.4. Combining the Circulating Levels of Selected Cytokines into a Logistic Regression Algorithm Created a Pattern Enabling the Recognition of Healthy People from Patients with NETs

**Figure 5:**
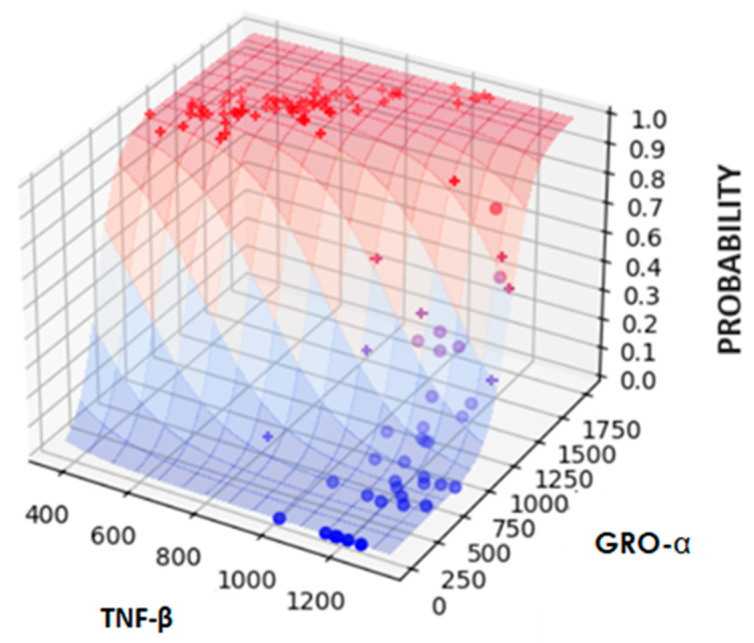
Three-dimensional plot of TNF-β (tumor necrosis factor β) and GRO-α (growth-regulated oncogene) variables. In this plot, the symbol “+” (in red) represents the results for patients classified as sick, while the symbol “o” (in blue) represents the results for healthy people. This visual representation helps in understanding the distribution and relationship of these variables in distinguishing between sick and healthy individuals. The plot demonstrates the clustering of data points, indicating how variations in TNF-β and GRO-α concentrations correlate with the probability of disease presence.

The multivariable logistic regression model with the feature elimination method identified a group of parameters whose changes allow for the greatest differentiation between patient groups. Variables were selected for the regression model using the hierarchical clustering method to minimize the number of predictors. The quality of the predictive regression model was checked with the use of the Leave-One-Out method. The obtained measures of the quality of precision of the performed multivariable logistic regression model were as follows: AUC, 0.987; sensitivity, 0.952; specificity, 0.976; accuracy, 0.95; and F1 score, 0.964. These measures show that the constructed regression is very good and can be used to select the best biomarkers. As a result of the performed analysis, two variables, namely TNF-β and GRO-α, were selected to predict the NEN occurrence. A three-dimensional graph illustrating the relationship between the TNF-β and GRO-α variables and the probability of classifying a patient as having the disease was generated, as shown in Figure 5. Additionally, the approximate shape of the hyperplane determining the model parameters was established. The model demonstrated an accuracy of 94.55%. To validate the model further, cross-validation was performed, confirming its robustness and predictive power. The inclusion of TNF-β and GRO-α as key variables in the model highlights their significant role in distinguishing between affected and unaffected individuals. These findings suggest that changes in TNF-β and GRO-α concentrations can serve as reliable biomarkers for neuroendocrine tumors, providing valuable insights for clinical diagnostics. The formula of the obtained regression model, which allows for the determination of the probability of confirming or excluding neuroendocrine tumors, is as follows:

$$p_{t}=\frac{1}{1+exp\left(-\left(-0.009171X_{t}+0.01051Y_{t}\right)\right)}$$

*p_t_*—probability of indicating the disease in the patient;
*X_t_*—measured value of the TNF-β parameter;
*Y_t_*—measured value of the GRO-α parameter.

## 4. Discussion

NENs are a unique group of cancers that, despite increasing physician awareness and greater access to endoscopic and imaging screening methods, still pose diagnostic challenges. The low incidence, heterogeneity of the tumors, and non-specific clinical presentations often complicate diagnosis [19]. International research conducted by Simron Singh et al. [20] in 2016 indicated an average delay in diagnosis from the onset of symptoms of 52 months. This is why markers supporting diagnosis and assessment of treatment effectiveness are being intensively sought.

The inflammatory microenvironment has been associated with the development and progression of NENs as an essential component of the tumor [14]. According to the research by Yu et al. [21], patients with inflammatory bowel disease had twice the risk of developing neuroendocrine tumors. Research in recent years has shown that GEP-NETs occur more frequently under conditions of chronic inflammation, and TNF-α, IL-6, IL-2, IL-1β, and TGF-β are particularly important for their development [22]. Due to the high vascularity of NENs, it can be postulated that angiogenic factors, such as VEGF, bFGF, PDGF, TGF-β, and SDF-1α+β, likely contribute significantly to the progression and angiogenesis in this type of cancer [23]. This thesis may be confirmed by our research, as all pro-angiogenic factors mentioned above, except SDF-1α+β, were statistically significantly increased in the patients with NENs compared to the control group, suggesting their involvement in the metastasis process. Among the analyzed chemokines, statistically significant changes were found in both pro-angiogenic and angiostatic factors. The synthesis and secretion of VEGF, one of the strongest factors inducing angiogenesis, has been found to be a characteristic feature of NEN cells [23]. Pavel et al. [24], in studies on patients with pancreatic, ileum, and bronchial carcinoid tumors, showed a statistically significant increase in VEGF concentration compared to the control group. VEGF levels were significantly higher in patients with disease progression compared to those with stable disease. Interestingly, the VEGF concentration increased significantly as the tumor grew but decreased significantly in stable disease, regardless of the type of therapy used. VEGF promotes tumor growth, particularly in low-grade, more differentiated NENs, because this process is unstable and heterogeneous in malignant tumors [15]. The neuroendocrine paradox is that higher VEGF concentrations in benign pancreatic neuroendocrine neoplasms (pNENs) are associated with a good prognosis, as vessel density determines the rate of differentiation rather than tumor aggressiveness [15,25]. PDGF-BB can stabilize newly formed vessels [26], and in the presented study, the value of this factor was significantly lower in the study group compared to the control group.

This study showed a significant increase in the concentration of both anti-inflammatory and pro-inflammatory interleukins in the study group compared to the control group. IL-2 is noteworthy as it plays a role in regulating the neuroendocrine system and the secretion of hormones in the gastrointestinal tract; it is also a means of inducing an anti-tumor response during tumor growth and progression [14,22]. In a study conducted by Pavel et al. [24], it was observed that the concentration of IL-8 was statistically significantly higher in the group of patients with NENs and in the group of patients with progressive disease compared to the control group, suggesting that this interleukin may be a predictor of shorter survival. These results propose VEGF and IL-8 as potential markers for prognosis and therapy control in NENs. PDGF-BB can stabilize newly formed vessels [26]. In this study, the value of this factor was significantly lower in all study groups compared to the control group. Tumor-derived PDGF is primarily responsible for angiogenesis, indirectly influencing tumor growth, metastasis, and drug resistance [27]. Its autocrine effect modulates malignant cell proliferation phenotypes, which may promote cancer [27]. Therefore, lower PDGF values may be beneficial for NENs, suggesting that PDGF or its receptor could be therapeutic targets. The excessive release of GRO-α can also lead to tissue damage, angiogenesis, and tumorigenesis. Cigrovski Berkovic et al. [16] demonstrated a relationship between TNF-α and IL-2 and the induction of GEP-NET. Those parameters were found to be more sensitive markers for both functional and non-functional GEP-NETs than the routinely used CgA. Similarly, IL-6 and IL-1β may be involved in the transformation of euroendocrine tissue [16]. Geisler et al. [28] performed similar analyses of a panel of 13 cytokines in 43 patients with confirmed NENs. For IL-1, IL-6, IL-8, IL-18, and TNF, the concentration was significantly higher, while for IL-10, the concentration was significantly lower compared to the control group. In most cases, our studies confirm these results; however, the concentration of IL-10, an anti-inflammatory parameter, was statistically significantly higher in the group of patients compared to the healthy group. In the case of TNF, the concentration of TNF-α was significantly higher, while the concentration of the cytotoxic cytokine TNF-β was significantly lower in the study group compared to the control group. There are no reports on the relationship between TNF-β and NENs, which requires further research focused on this factor. Buhrmann et al. [29] proved that TNF-β induces the proliferation and invasion of colorectal cancer cells comparable to TNF-α. In this study, the growth factors analyzed showed a statistically significant increase in their concentration in the serum of patients with NENs in each location compared to the control group. SCF, G-CSF, and β-NGF turned out to be some of the most sensitive markers among the studied variables. Interestingly, modifications in the expression of β-NGF may contribute to the progression of prostate NENs, and blocking its signaling pathways significantly inhibited the differentiation of this tumor [30]. However, in pancreatic carcinoid cell lines, NGF had mitogenic functions without affecting the cellular composition and their phenotype [31].

Increased CgA levels are found in neuroendocrine tumors, which is also confirmed by the results of this study. The cut-off value for CgA equal to 18.16060 ng/mL enabled discrimination between the control group and the patients, with a sensitivity of 72% and a specificity of 26%. The analysis carried out in this study indicates that the sensitivity of CgA is not the highest among the tested variables, and it may not be an ideal diagnostic parameter for NENs. However, research by other scientists proves its prognostic and predictive potential. The sensitivity of CgA in NENs depends on the primary site, degree of differentiation, and the advancement of the disease and ranges from 60 to 80% [32]. The highest CgA concentration in NENs is observed in carcinoid syndrome and in patients with liver metastases [33]. The increased level of CgA in NENs releasing this peptide may correlate with the tumor mass and recurrence, and it is considered a marker of poor prognosis and shorter survival in patients with GEP-NENs [34]. The average CgA level in small cell lung cancer (SCLC) is higher compared to the control group, while in the case of bronchial NENs, the CgA concentration is lower compared to other types of NENs and, importantly, may be comparable to the values found in other non-cancerous diseases [34]. Based on research conducted by Tsai et al. [35], it is concluded that CgA may be a predictive marker for tumor burden, overall survival, and tumor progression in GEP-NETs. Wang et al. [36] studied 145 patients with GEP-NETs and found that serum CgA levels were significantly higher in GEP-NEN patients with active disease compared with tumor resection patients or healthy subjects. They determined a CgA cut-off value of 95 ng/mL (sensitivity, 51.2%; specificity, 87.5%). Additionally, patients with serum CgA levels higher than 95 ng/mL had significantly shorter survival time compared with patients with CgA levels lower than this value. In their study, Zhang et al. [37] showed significantly higher CgA concentrations in patients with NENs compared to the control group with the cut-off point of 85.3 ng/mL (sensitivity, 64.4%; specificity, 92.7%). Zatelli et al. [38] determined the CgA concentration in 202 patients with GEP-NETs using two types of tests, namely immunoradiometric assay (IRMA) and ELISA, identifying the following cut-off points: IRMA: CgA = 53 ng/mL (sensitivity, 71.3%; specificity, 71%), and ELISA: CgA = 16 U/L (sensitivity, 84%; specificity, 85%). Lower CgA levels were found in patients with extensive metastases than in patients with liver metastases only. These studies confirm that CgA is not sensitive and specific enough to be an effective marker in the screening diagnosis of GEP-NETs. However, it can be used to monitor the patient, but it should be emphasized that CgA should be determined with the same type of test to ensure comparability of results.

This study demonstrated a positive correlation of several parameters, mainly inflammatory ones, with the CgA concentration, suggesting their usefulness both in diagnosis and in monitoring changes that occur during the disease progression or remission. The available literature confirms that CgA is involved in inflammation and autoimmune processes. It acts as a modulator of endothelial barrier function and an inhibitor of endothelial cell activation caused by inflammatory and pro-angiogenic cytokines, which are important in angiogenesis, inflammation, and carcinogenesis [34]. Some studies have indicated a positive correlation of CgA with procalcitonin and CRP or a negative correlation with certain interleukins (IL-1α and IL-1β) [17,39,40]. Analyzing parameters correlating with CgA is crucial to support the diagnostic value of this marker and to contribute to the rapid and effective identification of patients at high risk of metastatic disease at the time of initial diagnosis. However, due to the co-secretion of CgA with other bioactive peptides, there are many causes of increased CgA levels unrelated to NENs, which limits its sensitivity and specificity in diagnostic settings [41].

In this study, the determination of ROC curves allowed for the selection of parameters that best meet the criteria of markers for NENs. The parameters were chosen based on an AUC value of ≥0.9, indicating that they are strong markers of the inflammatory phenotype compared to others with lower diagnostic value. Highly sensitive parameters included TNF-β (93%), LIF (92%), IL-1α (92%), IL-16 (92%), GIP (90%), TNF-α (90%), β-NGF (90%), and G-CSF (90%), although the specificity of these parameters was low (≤5%). These results suggest that while high sensitivity can accurately diagnose the disease when present, the low specificity limits the ability to correctly exclude healthy individuals. For GRO-α, the sensitivity was 83% and specificity was 11%, whereas for TNF-β, these values were 93% and 5%, respectively. It should be noted that these variables, when used in the regression model, demonstrated the most desirable values in terms of both sensitivity and specificity.

Based on the ranking of the parameters (scheduled according to AUC), a correlation matrix was created. The logistic regression analysis allowed for the determination of the NEN prediction formula. This model was determined based on the concentrations of GRO-α and TNF-β, two parameters that distinctly differentiate this group of cancers. GRO-α is a pro-angiogenic factor that affects tissue damage and plays a role in cancer development. It functions both as a growth factor and a strong chemotactic factor, and it is involved in wound healing by influencing cell migration and angiogenesis [40]. Its expression is induced by PDGF, which may enhance the proliferation of cancer cells, as seen in glioma [42]. The role of GRO-α in primary tumor growth, angiogenesis, proliferation, and metastasis has been documented in several human cancers, including colorectal, breast, bladder, stomach, and melanoma [43]. TNF-β, on the other hand, is a cytotoxic factor with multiple effects on cells, including the processes of apoptosis, growth stimulation, and cell differentiation. Physiologically, it controls the development of lymphoid tissue and supports interactions between lymphocytes and their environment [44]. Importantly, the NEN diagnostic model presented in this study was constructed using the non-invasive parameters circulating in the bloodstream, which could significantly facilitate the verification of a larger number of patients with this type of cancer. There are certain limitations in the current studies, such as the lack of tumor stage categorization and some gaps in the medical history of the patients, as most of these cancers are diagnosed at the metastatic stage. Undoubtedly, to validate the stability and accuracy of the presented model, further research should be conducted on a larger cohort, but the use of the proposed markers seems to be promising. Other researchers have also developed models to facilitate the diagnosis of NENs. Wang et al. [45] established an insulinoma prediction model using multivariate logistic regression analysis that included BMI, glycated hemoglobin (HbA1C), glucose, and C-peptide. Similarly, Geisler et al. [28] developed a diagnostic index for NENs based on a logistic regression model combining IL-8, IL-10, and TNF to distinguish patients from healthy individuals. Undoubtedly, the use of the inflammatory marker panel, similar to the NETest, which could include the parameters mentioned above in combination with the commonly used CgA, might be proposed to significantly increase the sensitivity and specificity of the NEN diagnosis during clinical practice.

## 5. Conclusions

This study identified many new parameters that may participate in the diagnostic and potentially also prognostic or therapeutic processes in NENs. It has been shown that CgA is not a sufficiently reliable marker to be used independently in the diagnosis of neuroendocrine tumors. However, the simultaneous determination of other parameters correlated with CgA concentration could improve the diagnosis of NENs compared to the determination of each of these parameters separately. Undoubtedly, the direction of research aimed at constructing a model that allows for the effective identification of patients with NENs based on the concentration of several parameters seems very promising, given the diagnostic challenges associated with this type of cancer. Further multicenter studies on a larger group of patients are necessary to investigate the role of these new potential NEN markers, not only in diagnosis but also in disease progression and response to therapy.

## Figures and Tables

**Table 1:** Anthropometric and clinical characteristics of the patients with neuroendocrine neoplasms (NENs) and the control group. *p* < 0.05 was considered statistically significant.

Parameter		NENs	Control	*p*-Value
*n* (female/male)		84 (44/40)	40 (28/12)	-
Grade of the tumor		G1 64%/G2 36%	-	-
Body mass [kg]	Mean	78	68.5	0.1212
	SEM	2.559	1.772	
	Median	78	67	
	Q_1_; Q_3_	68.0; 87.0	60.0; 78.0	
Height [cm]	Mean	170	167	0.0507
	SEM	1.425	1.087	
	Median	170	165	
	Q_1_; Q_3_	164.0; 177.0	161.0; 171.0	
BMI [kg/m^2^]	Mean	26.9	24.6	0.1354
	SEM	0.754	0.513	
	Median	26.9	24.4	
	Q_1_; Q_3_	23.7; 29.1	22.4; 27.0	
Age [years]	Mean	58	57	0.1552
	SEM	2.043	1.48	
	Median	60.5	56.0	
	Q_1_; Q_3_	51; 66	50; 61	

Abbreviations used: BMI (body mass index), G1—well-differentiated tumor with low-grade malignancy; G2—intermediate-grade malignancy; Q_1_; Q_3_ (lower quartile; upper quartile); SEM (standard error of mean).

**Table 2:** Serum levels of BIO-Plex Pro Human Cytokine Screening Panel.

Parameter		NENs	Control	*p*-Value
Chromogranin A [ng/mL]	Mean	20.578	16.004	<0.001
	SEM	1.382	0.625	
	Median	21.068	15.690	
	Q_1_; Q_3_	17.466; 25.760	13.230; 18.191	
CTACK [pg/mL]	Mean	1927.558	894.803	<0.001
	SEM	113.570	48.462	
	Median	1801.195	842.840	
	Q_1_; Q_3_	1432.195; 2472.720	698.120; 1049.150	
Eotaxin [pg/mL]	Mean	165.514	102.969	<0.001
	SEM	12.692	9.032	
	Median	150.030	98.650	
	Q_1_; Q_3_	118.448; 194.305	64.200; 119.520	
bFGF [pg/mL]	Mean	101.890	29.309	0.002
	SEM	12.138	2.409	
	Median	133.220	26.730	
	Q_1_; Q_3_	3.260; 152.680	24.240; 31.070	
G-CSF [pg/mL]	Mean	737.933	34.778	<0.001
	SEM	125.906	4.791	
	Median	597.170	30.510	
	Q_1_; Q_3_	398.873; 876.375	6.350; 44.610	
GM-CSF [pg/mL]	Mean	21.652	0.480	<0.001
	SEM	6.058	0.000	
	Median	18.700	0.480	
	Q_1_; Q_3_	13.020; 24.770	0.480; 0.480	
GRO-α [pg/mL]	Mean	1201.284	591.017	<0.001
	SEM	41.437	61.061	
	Median	1203.480	636.390	
	Q_1_; Q_3_	1042.035; 1330.193	398.290; 866.195	
HGF [pg/mL]	Mean	603.779	290.862	<0.001
	SEM	45.835	16.337	
	Median	568.470	279.070	
	Q_1_; Q_3_	425.690; 721.720	228.550; 332.305	
IFN-α2 [pg/mL]	Mean	54.939	0.950	<0.001
	SEM	6.302	0.000	
	Median	47.600	0.950	
	Q_1_; Q_3_	33.820; 79.083	0.950; 0.950	
IFN-γ [pg/mL]	Mean	18.793	6.975	<0.001
	SEM	2.519	0.418	
	Median	15.540	6.220	
	Q_1_; Q_3_	11.360; 21.220	5.720; 8.480	
IL-1α [pg/mL]	Mean	132.769	4.536	<0.001
	SEM	19.067	0.562	
	Median	120.040	3.730	
	Q_1_; Q_3_	90.530; 156.540	3.730; 3.730	
IL-1β [pg/mL]	Mean	7.011	0.494	<0.001
	SEM	0.568	0.071	
	Median	7.430	0.290	
	Q_1_; Q_3_	6.160; 8.660	0.290; 0.290	
IL-1ra [pg/mL]	Mean	990.829	165.569	<0.001
	SEM	116.934	12.182	
	Median	982.710	147.050	
	Q_1_; Q_3_	687.760; 1221.310	127.355; 214.180	
IL-2 [pg/mL]	Mean	19.790	1.290	<0.001
	SEM	4.479	0.000	
	Median	1.290	1.290	
	Q_1_; Q_3_	1.290; 33.340	1.290; 1.290	
IL-2Rα [pg/mL]	Mean	196.963	46.435	<0.001
	SEM	21.452	3.012	
	Median	184.690	41.550	
	Q_1_; Q_3_	139.098; 237.415	34.695; 53.460	
IL-3 [pg/mL]	Mean	5.998	0.189	0.007
	SEM	3.151	0.059	
	Median	0.130	0.130	
	Q_1_; Q_3_	0.130; 0.130	0.130; 0.130	
IL-4 [pg/mL]	Mean	13.458	2.061	<0.001
	SEM	0.920	0.175	
	Median	13.380	2.110	
	Q_1_; Q_3_	11.430; 16.160	1.560; 2.630	
IL-5 [pg/mL]	Mean	104.324	7.597	<0.001
	SEM	18.354	3.967	
	Median	90.840	3.630	
	Q_1_; Q_3_	50.753; 135.880	3.630; 3.630	
IL-6 [pg/mL]	Mean	20.376	0.380	<0.001
	SEM	4.373	0.000	
	Median	16.450	0.380	
	Q_1_; Q_3_	10.828; 23.400	0.380; 0.380	
IL-7 [pg/mL]	Mean	29.907	1.920	<0.001
	SEM	4.014	0.000	
	Median	28.665	1.920	
	Q_1_; Q_3_	21.66; 39.41	1.920; 1.920	
IL-8 [pg/mL]	Mean	32.311	7.344	<0.001
	SEM	3.448	0.573	
	Median	27.120	6.170	
	Q_1_; Q_3_	19.71; 40.64	5.090; 8.835	
IL-9 [pg/mL]	Mean	553.179	503.224	0.06
	SEM	23.369	3.812	
	Median	529.595	507.130	
	Q_1_; Q_3_	461.395; 610.280	486610; 523.280	
IL-10 [pg/mL]	Mean	4.928	1.060	<0.001
	SEM	0.542	0.000	
	Median	5.810	1.060	
	Q_1_; Q_3_	3.650; 5.810	1.060; 1.060	
IL-12 (p40) [pg/mL]	Mean	120.544	14.680	0.004
	SEM	41.511	0.000	
	Median	14.680	14.680	
	Q_1_; Q_3_	14.680; 14.680	14.680; 14.680	
IL-12 (p70) [pg/mL]	Mean	8.943	1.430	<0.001
	SEM	2.805	0.000	
	Median	1.430	1.430	
	Q_1_; Q_3_	1.430; 13.280	1.430; 1.430	
IL-13 [pg/mL]	Mean	3.775	0.889	0.832
	SEM	2.599	0.283	
	Median	0.310	0.310	
	Q_1_; Q_3_	0.310; 0.310	0.310; 0.310	
IL-15 [pg/mL]	Mean	101.486	12.420	<0.001
	SEM	11.381	0.000	
	Median	98.040	12.420	
	Q_1_; Q_3_	76.918; 122.828	12.420; 12.420	
IL-16 [pg/mL]	Mean	142.684	46.065	<0.001
	SEM	10.309	3.278	
	Median	137.630	42.960	
	Q_1_; Q_3_	112.730; 167.280	33.995; 54.785	
IL-17A [pg/mL]	Mean	61.754	2.440	<0.001
	SEM	10.735	0.000	
	Median	45.020	2.440	
	Q_1_; Q_3_	2.440; 85.390	2.440; 2.440	
IL-18 [pg/mL]	Mean	79.345	37.177	0.031
	SEM	14.289	3.838	
	Median	54.020	33.040	
	Q_1_; Q_3_	23.450; 106.270	19.300; 46.660	
IP-10 [pg/mL]	Mean	491.863	505.567	0.163
	SEM	103.892	66.167	
	Median	342.245	404.620	
	Q_1_; Q_3_	259.760; 489.630	315.975; 498.360	
LIF [pg/mL]	Mean	289.931	13.931	<0.001
	SEM	20.797	2.315	
	Median	299.310	3.860	
	Q_1_; Q_3_	230.150; 345.485	3.806; 16.830	
MCP-1 [pg/mL]	Mean	135.349	35.973	<0.001
	SEM	9.869	3.654	
	Median	120.585	28.000	
	Q_1_; Q_3_	98.090; 168.800	2.077; 42.300	
MCP-3 [pg/mL]	Mean	9.159	0.480	<0.001
	SEM	1.112	0.000	
	Median	8.445	0.4800	
	Q_1_; Q_3_	4.178; 14.453	0.480; 0.480	
M-CSF [pg/mL]	Mean	26.649	5.488	<0.001
	SEM	6.974	0.547	
	Median	20.400	5.490	
	Q_1_; Q_3_	12.778; 30.860	3.510; 6.670	
MIF [pg/mL]	Mean	4393.315	551.077	<0.001
	SEM	1153.163	89.691	
	Median	2184.485	386.500	
	Q_1_; Q_3_	1205.510; 4484.038	283.140; 652.035	
MIG [pg/mL]	Mean	368.074	155.589	<0.001
	SEM	58.754	24.873	
	Median	289.300	107.630	
	Q_1_; Q_3_	181.283; 435.408	77.960; 182.010	
MIP-1α [pg/ml]	Mean	4.449	1.661	<0.001
	SEM	0.613	0.155	
	Median	4.500	1.450	
	Q_1_; Q_3_	0.120; 6.660	1.280; 1.900	
MIP-1β [pg/mL]	Mean	397.224	412.563	0.044
	SEM	14.943	4.009	
	Median	399.495	416.030	
	Q_1_; Q_3_	334.810; 428.508	400.110; 427.305	
β-NGF [pg/mL]	Mean	56.871	1.899	<0.001
	SEM	7.775	0.659	
	Median	51.175	0.470	
	Q_1_; Q_3_	33.278; 75.460	0.470; 0.470	
PDGF-BB [pg/mL]	Mean	2004.191	3439.245	<0.001
	SEM	159.215	263.637	
	Median	1815.200	2942.130	
	Q_1_; Q_3_	1436.980; 2339.930	2208.445; 4427.820	
RANTES [ng/mL]	Mean	19.621	12.113	<0.001
	SEM	1.092	0.881	
	Median	19.886	10.763	
	Q_1_; Q_3_	15.373; 26.467	9.172; 12.808	
SCF [pg/mL]	Mean	221.129	62.223	<0.001
	SEM	16.227	3.133	
	Median	205.960	59.320	
	Q_1_; Q_3_	154.495; 272.450	49.770; 73.340	
SCGF-β [ng/mL]	Mean	92.457	37.312	<0.001
	SEM	9.354	1.231	
	Median	79.978	38.600	
	Q_1_; Q_3_	54.132; 112.019	32.965; 42.116	
SDF-1α+β [ng/mL]	Mean	1.529	1.588	0.343
	SEM	0.053	0.051	
	Median	1.465	1.491	
	Q_1_; Q_3_	1.301; 1.715	1.383; 1.860	
TNF-α [pg/mL]	Mean	173.847	48.158	<0.001
	SEM	13.893	2.320	
	Median	163.640	45.940	
	Q_1_; Q_3_	140.030; 205.900	42.180; 50.255	
TNF-β [pg/mL]	Mean	723.991	1201.914	<0.001
	SEM	36.829	11.812	
	Median	685.300	1198.140	
	Q_1_; Q_3_	567.848; 825.223	1177.025; 1252.715	
TRAIL [pg/mL]	Mean	30.308	20.015	0.001
	SEM	3.599	0.979	
	Median	31.660	18.840	
	Q_1_; Q_3_	17.450; 42.550	17.225; 23.045	
VEGF [pg/mL]	Mean	1181.978	24.597	<0.001
	SEM	204.587	6.587	
	Median	1029.025	18.010	
	Q_1_; Q_3_	550.305; 1629.028	18.010; 18.010	

Abbreviations used: bFGF (basic fibroblast growth factor), β-NGF (beta-nerve growth factor), CTACK (cutaneous T-cell-attracting chemokine), Eotaxin (eosinophil chemotactic protein), G-CSF (granulocyte colony-stimulating factor), GM-CSF (granulocyte–macrophage colony-stimulating factor), GRO-α (growth-regulated alpha protein), HGF (hepatocyte growth factor), IFN-α2 (interferon alpha-2), IFN-γ (interferon gamma), IL-1α (interleukin 1 alpha), IL-1β (interleukin 1 beta), IL-1ra (interleukin 1 receptor antagonist), IL-2 (interleukin 2), IL-2Rα (interleukin 2 receptor alpha), IL-3 (interleukin 3), IL-4 (interleukin 4), IL-5 (interleukin 5), IL-6 (interleukin 6), IL-7 (interleukin 7), IL-8 (interleukin 8), IL-9 (interleukin 9), IL-10 (interleukin 10), IL-12 (p40) (interleukin 12 (p40)), IL-12 (p70) (interleukin 12 (p70)), IL-13 (interleukin 13), IL-15 (interleukin 15), IL-16 (interleukin 16), IL-17A (interleukin 17A), IL-18 (interleukin 18), IP-10 (interferon gamma-induced protein 10), LIF (leukemia inhibitory factor), M-CSF (macrophage colony-stimulating factor), MCP-1 (monocyte chemoattractant protein-1), MCP-3 (monocyte chemoattractant protein-3), MIF (macrophage migration inhibitory factor), MIG (monokine induced by gamma interferon), MIP-1α (macrophage inflammatory protein-1 alpha), MIP-1β (macrophage inflammatory protein-1 beta), PDGF-BB (platelet-derived growth factor-BB), RANTES (regulated on activation, normal T cell expressed and secreted), SCF (stem cell factor), SCGF-β (stem cell growth factor beta), SDF-1α+β (stromal cell-derived factor 1 alpha and beta), TNF-α (tumor necrosis factor alpha), TNF-β (tumor necrosis factor beta), TRAIL (TNF-related apoptosis-inducing ligand), VEGF (vascular endothelial growth factor). Parameters decreased in NENs are marked in red color.

**Table 3:** Analysis of serum cytokine in healthy control and patients with NETs. Receiver operating characteristic (ROC)/area under the curve (AUC) was used to indicate to a diagnostic marker with high sensitivity and specificity.

Parameter	AUC	AUC↓	AUC↑	*p*-Value	Cut-Off Point [pg/mL]	Sensitivity	Specificity
SCF	0.9575	0.9207	0.9942	<0.0001	>127.1400	0.8929	0.0000
G-CSF	0.9537	0.9142	0.9931	<0.0001	>161.5700	0.9048	0.0000
β-NGF	0.9513	0.9121	0.9906	<0.0001	>13.5500	0.9048	0.0286
IL-16	0.9505	0.9110	0.9899	<0.0001	>82.2400	0.9167	0.0286
MCP-1	0.9505	0.9123	0.9887	<0.0001	>82.7700	0.8571	0.0286
CgA	0.7485	0.6601	0.8369	0.0002	>18,160.60	0.7209	0.2571

Abbreviations used: β-NGF (nerve growth factor β), CgA (chromogranin A), G-CSF (granulocyte colony-stimulating factor), IL-16 (interleukin 16), MCP-1 (monocyte chemotactic protein 1), SCF (stem cell factor).

## Data Availability

The data presented in this study are available upon request from the corresponding author. The data are not publicly available due to privacy or ethical restrictions.
